# Editorial: Cytokine-Mediated Organ Dysfunction and Tissue Damage Induced by Viruses

**DOI:** 10.3389/fimmu.2020.00002

**Published:** 2020-01-22

**Authors:** Juliet V. Spencer, Piotr Religa, Michael H. Lehmann

**Affiliations:** ^1^Department of Biology, Texas Woman's University, Denton, TX, United States; ^2^Department of Medicine, Karolinska Institutet, Stockholm, Sweden; ^3^Institute for Infectious Diseases and Zoonoses, Ludwig-Maximilians-Universität München, Munich, Germany

**Keywords:** coxsackievirus, hantavirus, HCMV, HIV, influenza, MHV, RSV, TMEV

Cytokines are small proteins, mostly secreted into the extracellular environment, that bind to specific cell surface receptors, which mediate cell differentiation, migration, growth, and death. Gene expression and cellular release of cytokines are strictly regulated to assure proper function of cells, tissues, and organs. Upon virus infection, a cell starts producing type I interferons (IFN) and inflammatory cytokines (ICs) to restrict spread and replication of the respective virus. Ideally, the virus is completely eliminated by the immune system and the antiviral mechanisms are turned off within a reasonable time frame. However, there are different scenarios where this process does not work efficiently or does not happen at all, leading to cytokine-mediated organ dysfunction and tissue damage.

First, if a virus inhibits type I IFN production and signaling but does not prevent expression of ICs, then this virus spreads further, and more viral components are in the system, which continuously amplifies ICs production. The lung is one organ that is especially vulnerable to such a “cytokine storm,” triggered, for example, by infection with respiratory syncytial virus (RSV) or influenza virus. In a comprehensive review article, Bohmwald et al. describe in detail where and which cytokines are induced during human RSV infection and their potential contribution to damage of not only the lung but also the brain. The pathophysiological production of ICs is most probably also due to the ability of RSV to induce the expression of Toll-like receptor (TLR) 4 in human airway epithelial cells, which normally do not respond to endotoxin (1).

Aflatoxin B_1_ (AFB_1_), a mycotoxin produced by *Aspergillus flavus*, increases TLR4 expression as well as TLR2, IL-1β, and IL-6 expression in human monocyte-derived dendritic cells (2). Sun et al. confirmed upregulation of TLR4 expression by AFB_1_ in porcine alveolar macrophages (PAMs) and mice infected with swine influenza virus (SIV) subtype H1N1. Importantly, they show that AFB_1_ exacerbates lung damage in mice during SIV infection, caused by a TLR4-dependent increase in viral replication and TNF levels. Consequently, uptake of AFB_1_, for example by contaminated food (3), could aggravate the course of flu.

Experimental evidence suggests that extrarespiratory induction of ICs such as TNF, IL-6, and IL-8 contributed to deadly infection with the 1918 H1N1 influenza A virus (IAV) strain (4), which hit mankind during World War I, a period when not sufficient food was available (5). Similarly, systemic high levels of IL-6 and IL-8 were detected in humans infected with IAV subtype H5N1, especially in those with fatal outcome, but not in humans infected with IAV subtype H3N2 or H1N1 (6). Hence, it appears that an IAV which just passed the animal-human barrier is much more harmful than a human adapted strain. In this respect, Krischuns et al. discovered that infection of human alveolar epithelial cells with highly pathogenic avian influenza virus (HPAIV) strains, but not with human adapted IAV strains, leads to constitutive phosphorylation of tripartite motif (TRIM) 28 at S473 and increased production of IFN-β, IL-6, and IL-8. TRIM28 negatively regulates transcription in a number of ways (7), and the inability of the non-human adapted influenza strains to prevent its deactivation could well explain the dysregulated cytokine expression observed in individuals infected with the IAV subtype H5N1 or the 1918 pandemic H1N1 IAV. In conclusion, severe organ damage can occur if a virus passes from its natural host to another species where it can or gains the ability to replicate without having an effective mechanism to block immunity of the other species. such a scenario is frequently observed in humans when they are infected with hantavirus (8). Possibly, activation of bystander CD8+ T cells after hantavirus infection, as shown in an original article by Raftery et al., could be a reason for the organ damages in humans. Indeed, viral-activated bystander memory CD8+ T cells can cause organ damage in a T cell receptor independent manner (9). In contrast, no apparent disease is observed in rodents, the natural reservoir hosts of all hantavirus species, which is similar to humans where a lifelong, persistent infection with human cytomegalovirus (HCMV) remains normally inconspicuous. However, there are several situations in which HCMV induces damage to organs and tissues, and Clement and Humphreys review the involvement of cytokines therein.

The cellular tropism of human and simian immunodeficiency virus is limited, but dysfunctions and damages also occur in the hearts, lungs, kidneys, and brains of infected individuals. Lehmann et al. provide a model to understand the complexity of these pathologies based on recent cell biology findings about systemic distribution of the viral Nef protein and improved understanding of C-C motif chemokine ligand 2 (CCL2) dependent transendothelial cell migration. The focus of this Mini Review is on the brain, where non-physiological induction of CCL2 expression not only drives encephalitis but also affects signaling and survival of neuronal cells. Indeed, antiviral and anti-inflammatory cytokine expression have to be well-coordinated during a viral infection in order to enable elimination of the pathogen on the one hand, and to avoid tissue and organ damage on the other hand. This problem is discussed by Savarin and Bergmann on the basis of a murine model of encephalomyelitis induced by a neurotropic strain of mouse hepatitis virus where the interplay of IFN and IL-10 dictates the extent of viral control and tissue pathology.

Type I IFN is required to restrict coxsackievirus B3 (CVB3) replication (10), and Liu et al. found that functional TRIM21 is important for high level type I IFN expression *in vitro* and *in vivo*. Additionally, they show that TRIM21 deficiency leads to higher viral titers, stronger cardiac and pancreatic damages, and higher levels of ICs including CCL2 in mice infected with CVB3. Meyer et al. detected increased levels of colony stimulating factor 1 (CSF-1) in heart biopsies of patients with myocarditis, and by using nanoparticle-encapsulated siRNA directed against CSF-1 they could decrease CVB3-induced monocyte infiltration and heart damage in mice. Beling and Kespohl suggest that therapeutic targeting the proteasome could help to prevent immunopathology of the heart, which can be triggered by many different viruses. Theiler's murine encephalitis virus (TMEV) induces myocarditis in mice, but this virus can also induce demyelination of neurons depending on the mouse strain. A Hypothesis and Theory article by Omura et al. summarizes findings about the different nature of these diseases and provides evidence that TMEV induces cell-type specific innate immune responses and distinct organ-specific pathology. Thus, the choice of the right animal model to study virus induced immunopathology can be challenging. For that, Manickam et al. provide a thorough review of non-human primate models for understanding the extent of cytokine-mediated tissue damage during many different types of virus infection, including dengue virus, HCMV, hepatitis B and C virus, HIV, influenza virus, and Zika virus.

Collectively, this Research Topic introduces some of the complex virus-host interactions that can tip the scales toward immunopathology. The common themes that emerge from this collection include the potential for use of cytokines as markers of disease and the manipulation of certain cellular molecules as therapeutic options.

## Author Contributions

All authors listed have made a substantial, direct and intellectual contribution to the work, and approved it for publication.

### Conflict of Interest

The authors declare that the research was conducted in the absence of any commercial or financial relationships that could be construed as a potential conflict of interest.
